# Microbial community composition of terrestrial habitats in East Antarctica with a focus on microphototrophs

**DOI:** 10.3389/fmicb.2023.1323148

**Published:** 2024-01-05

**Authors:** Ekaterina Pushkareva, Josef Elster, Sakae Kudoh, Satoshi Imura, Burkhard Becker

**Affiliations:** ^1^Department of Biology, Botanical Institute, University of Cologne, Cologne, Germany; ^2^Institute of Botany, Academy of Sciences of the Czech Republic, Třeboň, Czechia; ^3^Centre for Polar Ecology, University of South Bohemia, České Budějovice, Czechia; ^4^Department of Polar Science, The Graduate University for Advanced Studies, SOKENDAI, Tachikawa, Japan; ^5^National Institute of Polar Research, Research Organization of Information and Systems, Tachikawa, Japan

**Keywords:** East Antarctica, terrestrial environment, amplicon sequencing, cyanobacteria, eukaryotic microalgae

## Abstract

The Antarctic terrestrial environment harbors a diverse community of microorganisms, which have adapted to the extreme conditions. The aim of this study was to describe the composition of microbial communities in a diverse range of terrestrial environments (various biocrusts and soils, sands from ephemeral wetlands, biofilms, endolithic and hypolithic communities) in East Antarctica using both molecular and morphological approaches. Amplicon sequencing of the 16S rRNA gene revealed the dominance of Chloroflexi, Cyanobacteria and Firmicutes, while sequencing of the 18S rRNA gene showed the prevalence of Alveolata, Chloroplastida, Metazoa, and Rhizaria. This study also provided a comprehensive assessment of the microphototrophic community revealing a diversity of cyanobacteria and eukaryotic microalgae in various Antarctic terrestrial samples. Filamentous cyanobacteria belonging to the orders Oscillatoriales and Pseudanabaenales dominated prokaryotic community, while members of Trebouxiophyceae were the most abundant representatives of eukaryotes. In addition, the co-occurrence analysis showed a prevalence of positive correlations with bacterial taxa frequently co-occurring together.

## Introduction

Antarctica is located in the Southern Hemisphere and considered as the most extreme place in the world. Ice-free area constitutes around 1% of whole continent and mainly confined to coastal margins (Cowan, 2014). Organisms inhabiting ice-free regions of Antarctica are adapted to extreme environmental conditions (e.g., low temperatures, high solar radiation, frequent freeze–thaw cycles, limited nutrients availability, desiccation, and osmotic stress). They develop various strategies enabling to grow and thrive in harsh Antarctic environment. The limited availability of liquid water is one of the most important factors controlling the growth and development of terrestrial microbiota. The water necessary for Antarctic terrestrial microbiota is only available during the short austral summer (Cary et al., 2010).

Terrestrial environments in Antarctica include soils, biocrusts, rocks, mats, sediments, and other habitats that harbor diverse microbial communities. Their composition greatly varies from one to another locality even within short distances (Almela et al., 2021). However, same microorganisms can be observed in different areas and ecosystem types unless the habitat is isolated (Doytchinov and Dimov, 2022). Most of the available studies about Antarctic terrestrial ecosystem are focused on soil microbiota (Wang et al., 2015; Almela et al., 2021). Recently, assemblies of organisms (cyanobacteria, algae, fungi, mosses, lichens etc.) that colonize the soil surface were determined as biocrusts (biological soil crusts). These biocrusts have higher abundance and diversity of microorganisms compared to the underlying soils (Bastida et al., 2014). Furthermore, photosynthetic activity in the soil profile was observed in the first 5–10 mm of the soil surface, demonstrating the presence of established photosynthetic microorganisms in biocrusts (Pushkareva et al., 2016). These microorganisms are crucial for carbon (C) and nutrient cycling, particularly in Antarctica, where only two vascular plant species are present. In general, bacterial communities of Antarctic soils and biocrusts are predominantly comprised of Actinobacteria, Acidobacteria, Bacteroidetes, Chloroflexi, Cyanobacteria, Gemmatimonadetes, and Proteobacteria (Bajerski and Wagner, 2013; Zhang et al., 2020). Antarctic terrestrial eukaryotic microbiota are mainly represented by Alveolata, Chloroplastida, Fungi and Metazoa (Obbels et al., 2016; Zhang et al., 2020). Yet, other terrestrial habitats in Antarctica received far less attention. For example, microbial communities that thrive on, under, or within rocks are prevalent in Antarctica. These lithic communities are usually dominated by either cyanobacteria or eukaryotic microalgae (De Los Ríos et al., 2014). They acquire photoprotective mechanisms and produce extracellular polymeric substance (EPS), enabling them to survive in such extreme environment.

Studies on Antarctic terrestrial microorganisms have mainly been conducted in Antarctic Peninsula or Antarctic Dry Valleys and the majority of continental Antarctica has received limited attention due to the difficulty of access. As a result, our understanding of the microorganisms inhabiting Antarctica is fragmented. Here, we applied molecular and morphological methods to study microbial diversity and abundance in various terrestrial environments in the Enderby Land and Queen Maud Land, Antarctica. In addition, a detailed analysis of the microphotrophic community composition was performed. The following questions were addressed: (i) what are the microorganisms inhabiting terrestrial environment in the East Antarctica? (ii) what are the main microphototrophic genera in these environments? and (iii) which microorganisms co-occur together in this extreme continent?

## Materials and methods

### Localities description and sampling

**Figure 1:**
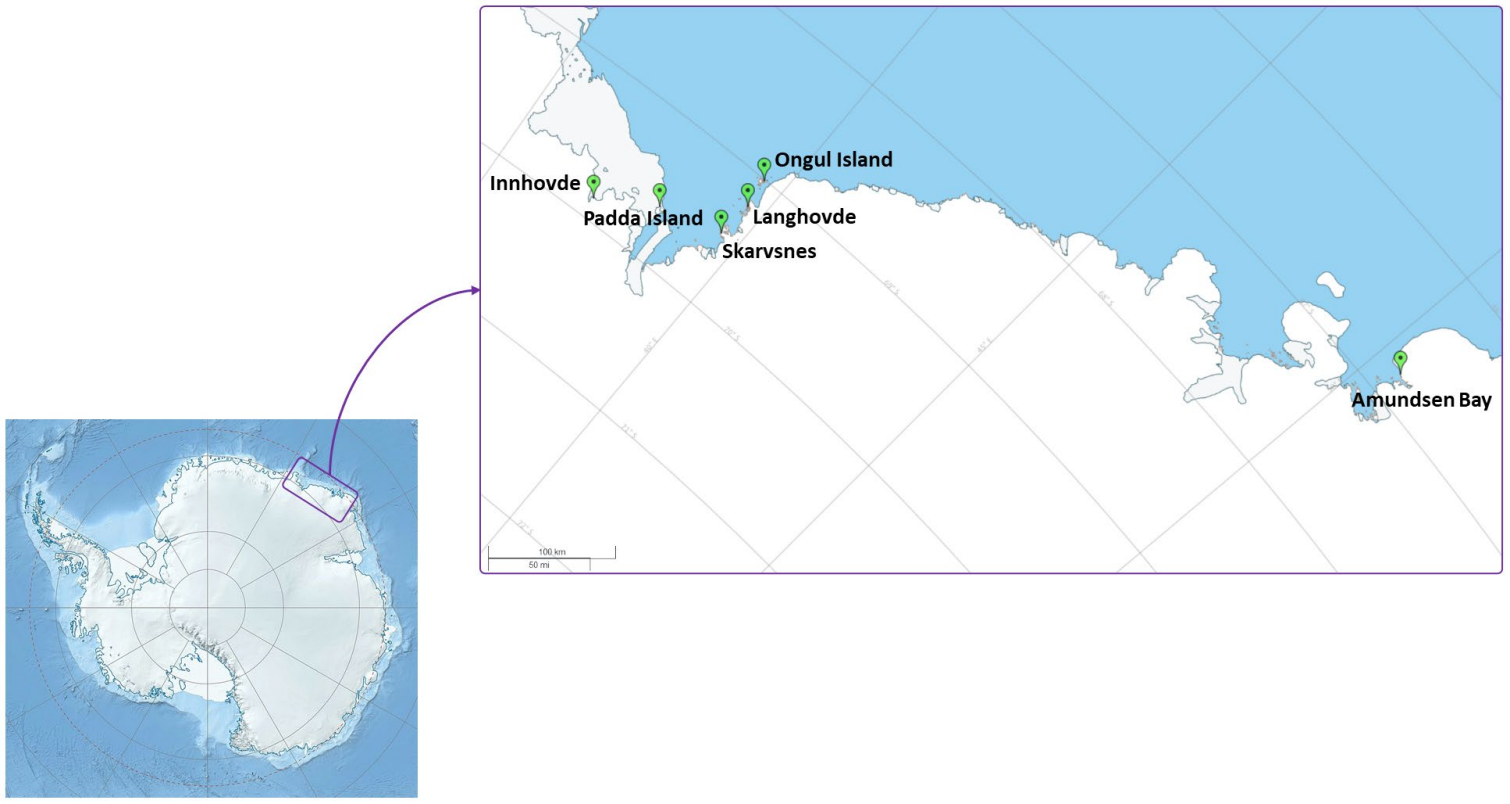
Map of the sampling localities.

Enderby Land and Queen Maud Land located in East Antarctica were visited in austral summer of 2018/2019 during Japanese Antarctic Research Expedition (JARE60). The coldest month in recorded in Syowa station is August with a mean air temperature of around −19°C and the warmest is January with a mean air temperature of around −1°C. The most of the territory is covered by the ice sheet. The map of the sampling sites and samples descriptions are present in Figure 1 and Table 1, respectively. The samples with visible algae or cyanobacteria biomass were collected from various terrestrial habitats (Supplementary Figure S1):

**Table 1:** Description of the samples in continental Antarctica.

Sample	Description	Location	GPS coordinates	
			S	E
Amu1	Biocrust in the rookery	Amundsen Bay	66°47′28”	50°33′06”
Amu2	Biocrust near the rookery		66°47′26”	50°32′58”
Amu5	Biomass on penguin feathers surface		66°47′23”	50°33′22”
Amu8	Biocrust		66°47′22”	50°33′27”
Amu9	Biofilm on rocks		66°47′23”	50°33′45”
Amu14	Biocrust with penguin feathers		66°47′20”	50°33′49”
In1	Biofilm on rocks	Innhovde	69°51′22”	37°06′29”
Lang4	Black spots with mosses	Langhovde	69°14′27″	39°43′37”
Lang7	Biocrust next to the stream		69°14′28”	39°44′29″
Lang21	Black biocrust		69°14′43”	39°43′29”
Lang35	Mineral soil		69°14′28”	39°44′19”
Lang36	Lichens on the moss biocrust		69°14′28”	39°44′19”
Lang37	Black biocrust with lichens		69°14′28”	39°44′19”
Lang41	Frost boil soil		69°14′72”	39°43′51”
Lang46	Endolithic community		69°14′18”	39°43′34”
Pad1	Black biocrust with moss	Padda Island	69°37′01”	38°16′25”
Pad2	Poor biocrust		69°37′01”	38°16′25”
Pad3	Sand from ephemeral wetlands		69°36′51	38°16′21”
Skar18	Black biocrust	Skarvsnes	69°29′22”	39°35′15”
Skar19	Biocrust in the rookery		69°29′36”	39°33′43
Skar22	Biomass on surface of seal mummy		69°27′44”	39°35′00”
Skar35	Sand from ephemeral wetlands		69°28′27”	39°38′44”
Skar38	Hypolithic community		69°28′51”	39°39′15”
Syo4	Biocrust	Ongul Island (Syowa station)	69°00′47”	39°35′05”
Syo6	Black biocrust		69°00′50”	39°34′11”

1. Amundsen Bay is a 39-km embayment in Enderby Land. Sampling sites were located near the two penguin rookeries: Adélie penguin (*Pygoscelis adeliae*) and Emperor penguin (*Aptenodytes forsteri*). Six samples (Amu) were collected. The surroundings of 4 samples were influenced by penguin guano or seasonal penguin presence.
2. Innhovde Point (In) is a rock point located along the shore of Fletta Bay, Queen Maud Land. It is barren area surrounded by ice cap and one biofilm sample (In) was collected.
3. Langhovde Hills are bare rocky hills along the eastern shore of Lützow-Holm Bay, Queen Maud Land, where 8 samples (Lang) were taken. The samples included different types of biocrusts, soils, and endolithic community.
4. Padda Island lies near the west side of the entrance to Havsbotn in Lützow-Holm Bay, Queen Maud Land. The island represents rocky barren habitat and the three collected samples (Pad) were either biocrusts or sands from ephemeral wetlands.
5. Skarvsnes Foreland, located in the east part of Lützow-Holm Bay, Queen Maud Land, is an extensive area with bare rock peaks. A total of 5 samples (Skar) were collected including biocrusts, biomass from the seal mummy surface, sand from ephemeral wetlands as well as hypolithic community.
6. Syowa Station is a Japanese research station located in the northern part of the Ongul Island in Queen Maud Land, Antarctica. Two biocrust samples (Syo) with precipitated salts were collected there.

### Soil analysis

Chemical analyses of two technical replicates per each sample were performed according to Czech and European Union standards (ISO 10390, ISO 10523, ČSN EN 27888, ISO 11465, ČSN EN ISO 11732, ČSN EN ISO 13395, and ČSN EN ISO 15681–1). Conductivity (μS cm^−1^) and pH were evaluated in demineralized and distilled water, respectively. The samples were further dried at 105°C to constant weight and then combusted at 450°C. Soil organic matter content (SOM) was calculated as difference between two weights. Total phosphorus (TP) was estimated using spectrometric determination of phosphorus soluble in sodium hydrogen carbonate solution. Mineral nitrogen (N_mineral_) was calculated as a sum of N–NH_4_, N–NO_3_ and N-NO_2_ concentrations, which were measured using a QuikChem®8500FIA automated ion analyzer (LachatInstruments, Loveland, USA).

A few samples were partially (Amu9, Lang7, Lang21, Lang35, Lang36, Skar38) or not (Lang46 and Skar22) analyzed due to the lack of material.

### Cell number and biovolume of microbial phototrophs

Cell number and biovolume of cyanobacteria and eukaryotic microalgae in the biocrusts was estimated by light and epifluorescence microscopy (BX51, Olympus) using two technical replicates as for chemical analysis. A non-staining method was employed using chlorophyll autofluorescence according to Kaštovská et al. (2005). Biocrust sample (1 g) was suspended in 4 mL of distilled water and mixed thoroughly. A total of 20 μL of this suspension was used for the microscopy. Cyanobacteria and eukaryotic microalgae were quantified using filter cubes (Olympus, MWG) with green excitation at 510–550 nm (emission 590 + nm) and blue excitation at 450–480 nm (emission 515 + nm), respectively. Four measurements were conducted for each technical replicate. Basic geometric equations for cylinders with hemispherical ends and spheres were applied to calculate cell biovolume (Hillebrand et al., 1999).

### DNA isolation and amplicon sequencing

A total DNA was extracted from each sample (0.3 g) in four technical replicates using the DNeasy PowerSoil Pro Kit (QIAGEN, USA) according to the manufacturer’s instructions. The extracted DNAs were then sent to the Microsynth AG (Balgach, Switzerland), where two-step PCR and amplicon sequencing of 16S and 18S rRNA genes using Illumina MiSeq platform (v2, 2 × 250 bp) were performed. Two sets of primers were used in this study: for bacteria (341F – CCTACGGGRSGCAGCAG, 802R – TACNVGGGTATCTAATCC) amplifying V3–V4 region of the 16S rRNA gene, and for eukaryotes (tarEuk_F – CCAGCASCYGCGGTAATTCC, tarEuk_R – ACTTTCGTTCTTGATYRA; Stoeck et al., 2010) targeting V4 region of the 18S rRNA gene. The raw reads were submitted to the Sequence Read Archive (SRA) under the projects PRJNA936101 and PRJNA936193 for 16S and 18S rRNA sequencing, respectively.

### Bioinformatic and statistical analyses

The bioinformatic procedure was performed following the recommended protocol of USEARCH (Edgar, 2013). Obtained Illumina paired-end reads were merged using USEARCH (version 11.0.667) with a minimum length of 220 bp. Furthermore, reads were quality filtered using VSEARCH (version 2.14.1) (Rognes et al., 2016) and, subsequently, clustered into amplicon sequence variants (ASV) following UNOISE algorithm implemented in USEARCH with default settings. ASV taxonomic assignments were conducted based on SILVA database (version 138.1). Additionally, ASVs classified as chloroplasts, mitochondria, and Archaea were discarded from the bacterial data set.

All statistical analyses were performed in R (version 4.1.3). Alpha diversity indices (ASV richness, Chao1, Evenness, and Shannon’s diversity) were calculated using the package *vegan* (Oksanen, 2013). The differences in parameters among sampling sites and regions were tested with one-way analysis of variance (ANOVA) and Tukey’s HSD *post hoc* test. A value of *p* <0.05 was considered significant in all statistical tests. Pearson correlation test between soil chemistry and relative abundance of different microbial taxa was also performed. Community dissimilarities among the sampling sites and three regions based on number of reads obtained by amplicon sequencing were assessed by non-metrical multidimensional scaling (NMDS) using the package *phyloseq* and permutational multivariate analysis of variance (PERMANOVA).

In addition, a co-occurrence network was built using the package *cooccur* (*true_rand_classifier* = 0.2) with taxonomical classification at the order or class level.

## Results

### Chemical characteristics

Chemical characteristics of the samples are present in Table 2. In brief, pH of the measured samples was in a range of 5.19–8.35. Biocrusts from the rookery (Amu1 and Skar19) had higher SOM, N_mineral_ and TP contents than the other samples. Likewise, biomass on penguin feathers surface (Amu5) had high SOM and N_mineral_ contents. Frost boiled soil (Lang41) had slightly alkaline pH but very low SOM, N_mineral_ and TP contents.

**Table 2:** Chemical characteristics of the Antarctic samples.

Sample	pH (H_2_O)	Conductivity, μS/cm	SOM, %	N_mineral_, %	TP, %
Amu1	7.01	8,230	20.30	9.21	4.54
Amu2	7.83	3,220	1.48	0.28	0.08
Amu5	5.91	586	17.81	2.85	0.71
Amu8	5.71	113	8.92	0.69	0.18
Amu9	5.46	1838	NA	NA	NA
Amu14	5.19	675	5.86	0.46	0.08
In1	6.92	80	5.04	0.43	0.20
Lang4	7.11	261	1.00	0.06	0.20
Lang7	5.93	215	NA	NA	NA
Lang21	7.73	5,010	NA	NA	NA
Lang35	6.58	139	NA	NA	NA
Lang36	NA	NA	0.60	0.08	0.31
Lang37	6.26	109	1.70	0.67	0.40
Lang41	8.35	2,700	0.62	0.09	0.17
Lang46	NA	NA	NA	NA	NA
Pad1	5.57	119	2.23	0.17	0.24
Pad2	6.31	34	1.29	0.12	0.29
Pad3	6.60	141	1.39	0.31	0.32
Skar18	7.64	504	3.26	0.09	0.13
Skar19	7.53	2080	13.29	7.58	4.22
Skar22	NA	NA	NA	NA	NA
Skar35	8.19	465	2.71	0.03	0.15
Skar38	7.17	284	NA	NA	NA
Syo4	7.55	4,420	1.11	0.09	0.26
Syo6	6.55	274	0.66	0.34	0.21

SOM, N_mineral_, and TP correspond to soil organic matter, mineral nitrogen and total phosphorus, respectively. NA indicates that measurements are not available for the sample.

### Microbial community composition

**Figure 2:**
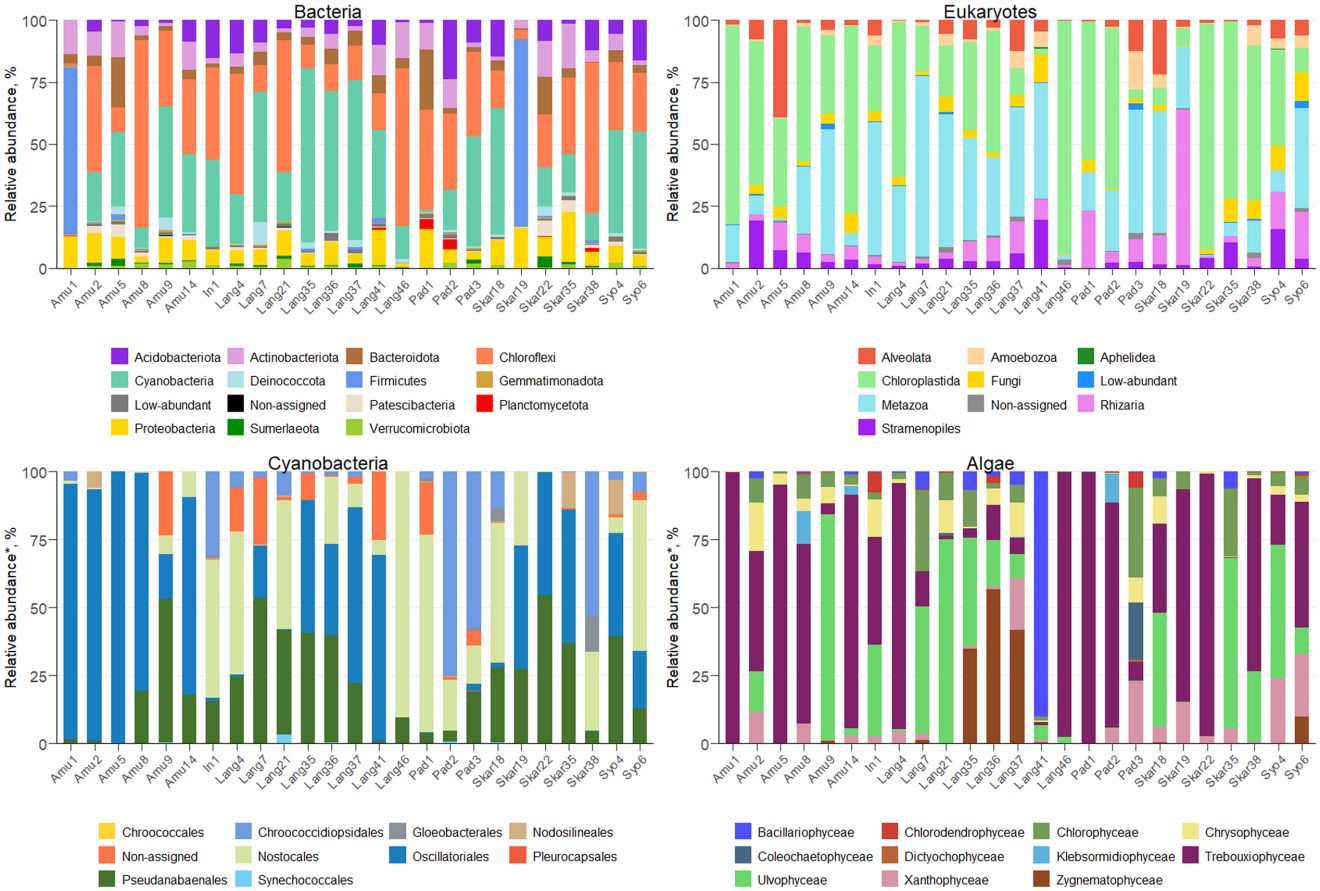
Microbial profiles in terrestrial Antarctic samples.

Amplicon sequencing using general bacterial primers resulted in 1.5 M quality filtered reads and 5,013 ASVs assigned to bacteria. ASV richness in the sites varied from 53 to 716 bacterial ASVs and from 48 to 618 eukaryotic ASVs (Supplementary Figure S2). The majority of bacterial reads were assigned to Chloroflexi (1,139 ASVs), Cyanobacteria (365 ASVs) and Firmicutes (132 ASVs), depending on the sample (Figure 2). Amplicon sequencing using eukaryotic primers produced 5 M reads clustered into 2,184 ASVs. Eukaryotic community was dominated by Chloroplastida (284 ASVs), Alveolata (278 ASVs), Rhizaria (544 ASVs) and Metazoa (99 ASVs). In total, 375 bacterial and 246 eukaryotic genera were identified across the samples.

The majority of the collected samples were dominated by Cyanobacteria and Chloroplastida and/or Metazoa (Figure 2). Chloroplastida was the most abundant eukaryote in the majority of the samples from Amundsen Bay, Skarvsnes and Padda Island, while Metazoa dominated more samples from Langhovde and Innhovde. The dominance of both Cyanobacteria and Metazoa was observed in biofilms (Amu9 and In1), several biocrusts (Lang7, Lang37 and Skar18), soils (Lang35 and Lang 41) and sand (Pad3). Furthermore, biocrusts collected in the rookeries (Amu1 and Skar19) were dominated by Firmicutes within bacteria and either Chloroplastida (Amu1) or Rhizaria (Skar19) within eukaryotes.

**Figure 3:**
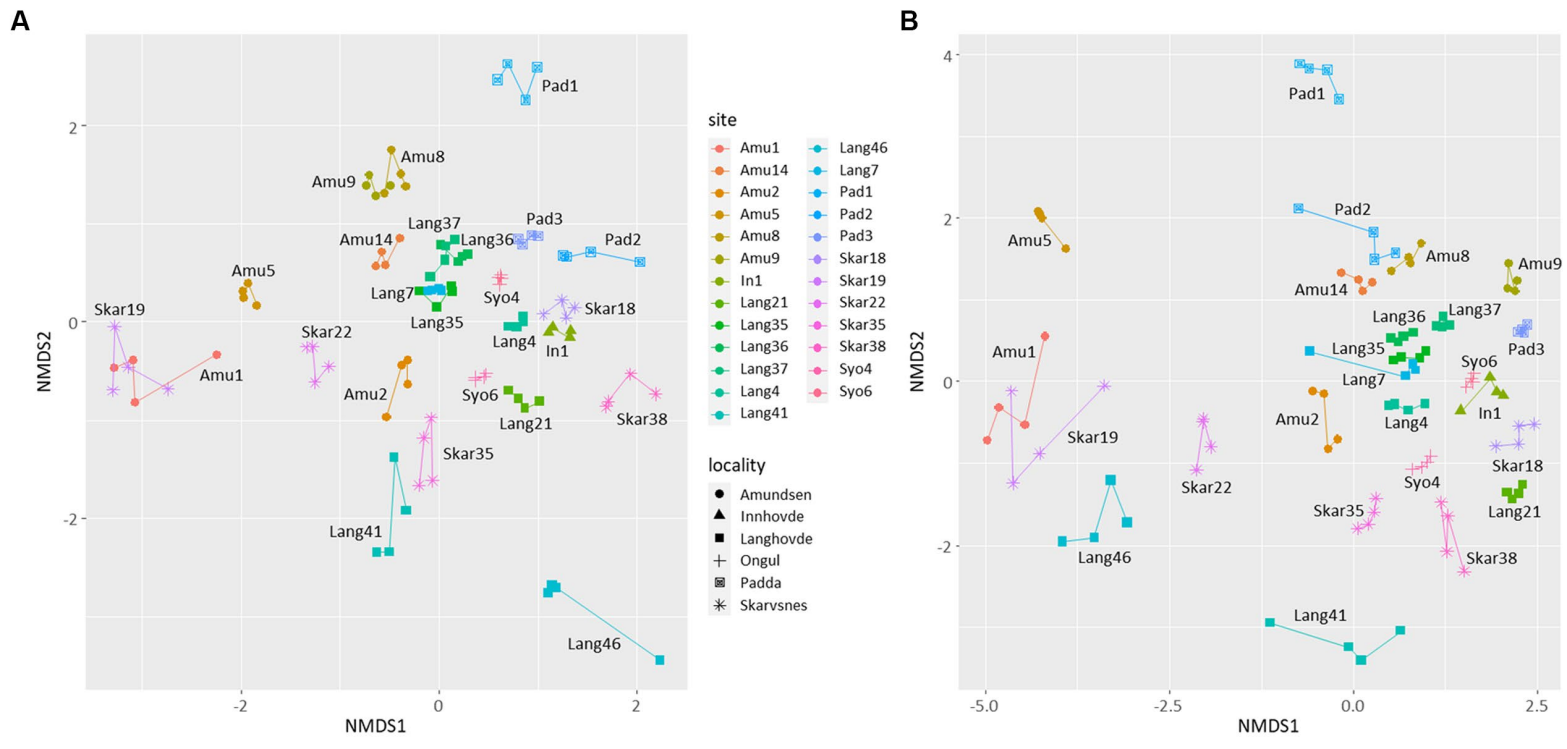
Non-metric multidimensional scaling (NMDS) plots based on the bacterial **(A)** and eukaryotic **(B)** reads. Lines connect four replicates from each site.

NMDS plot showed that the replicates were similar to each other and grouped by the site of their origin (Figure 3). Furthermore, the microbial diversity among the sites was significantly different based on PERMANOVA analysis (*p* < 0.05 for both 16S and 18S datasets). There were no clear clusters based on the locality or type of the samples with some exceptions. For example, few samples from Langhovde (Lang7, Lang35, Lang36, Lang37) and Amundsen Bay (Amu8, Amu9 and Amu14) were closely related to each other within the locality of origin. Besides, biocrusts from the rookeries (Amu1 and Skar19) were also similar to each other.

In addition, there were no significant correlations between the microbial diversity and soil parameters with two exceptions. Relative abundance of Firmicutes was strongly positively correlated with total P and N (*r*^2^ = 0.99 and 0.96, respectively; *p* < 0.05).

### Composition and abundance of microbial phototrophs

A total of 279 ASVs were assigned to Cyanobacteria (Figure 2; Supplementary Table S1) and 26 genera were identified. The majority of the samples were dominated by Oscillatoriales (22 ASVs) and 6 genera were recorded (*Coleofasciculus*, *Geitlerinema*, *Kamptonema*, *Oscillatoria*, *Tychonema*, *Wilmottia*). ASV11 assigned to *Wilmottia* constituted 47 and 64% of total cyanobacterial reads in the two samples (Amu8 and Amu14, respectively). Likewise, ASV18, identified as *Tychonema*, comprised 67 and 66% of total cyanobacterial reads in Amu1 and Amu5, respectively, but was not found in the majority of the samples. Another filamentous order Pseudanabaenales (114 ASVs) had high abundance in the samples and the majority of assigned ASVs belonged to either *Leptolyngbya* (17 ASVs) or *Phormidesmis* (42 ASVs). Order Nostocales was presented by 8 genera (*Calothrix*, *Loriellopsis*, *Mastigocladopsis*, *Nodularia*, *Nostoc*, *Petalonema*, *Rivularia*, *Scytonema*) and prevailed in the 7 samples (In1, Lang4, Lang21, Lang 46, Pad1, Skar18 and Syo6). Besides, 90% of cyanobacterial reads in Lang46 belonged to ASV35 assigned to Nostocales, but not identified beyond the order level. This endolithic sample (Lang46) had only 12 cyanobacterial ASVs and was very distinct from the other samples. Furthermore, three samples (Pad2, Pad3 and Skar 38) were dominated by Chroococcidiopsidales and two genera (*Aliterella*, *Chroococcidiopsis*) were identified. In addition, Synechococcales, in particular *Acaryochloris* and *Synechococcus*, were observed in the 9 samples.

Eukaryotic microalgae comprised from 3 to 80% of the total eukaryotic reads, depending on the sample (371 ASVs in total; Figure 2; Supplementary Table S1). The dominant microalgae in the majority of the samples were Trebouxiophyceae (149 ASVs). ASV2 assigned to *Prasiola* dominated algal community in the samples Amu1, Amu5 and Skar22. Besides, 90 ASVs from Trebouxiophyceae were identified at the genus level (20 genera; Supplementary Table S1). Furthermore, class Chlorophyceae prevailed in the sample Pad3 (33% of total algal reads) and 15 genera were identified across all the samples (Supplementary Table S1). Ulvophyceae dominated in seven samples (Amu9, Lang7, Lang21, Lang35, Skar18, Skar35, Syo4) and four different genera were identified, namely *Desmochloris*, *Scotinosphaera*, *Acrosiphonia*, *Pseudendoclonium*. The only one ASV from Zygnematophyceae, genus *Cosmarium*, exerted dominance in Lang36 and Lang37. Moreover, the sample Lang41 had 90% of total algal reads belonging to Bacillariophyceae with majority of reads assigned to genus *Luticola*. Other diatom ASVs identified at the genus level included *Chaetoceros*, *Fistulifera*, *Hantzschia*, *Humidophila*, *Parlibellus*, *Pinnularia*. In addition, several genera were identified within class Xanthophyceae, namely *Botrydiopsis*, *Chlorellidium*, *Mischococcus*, *Pleurochloris*, *Pseudopleurochloris* and *Xanthonema*.

**Figure 4:**
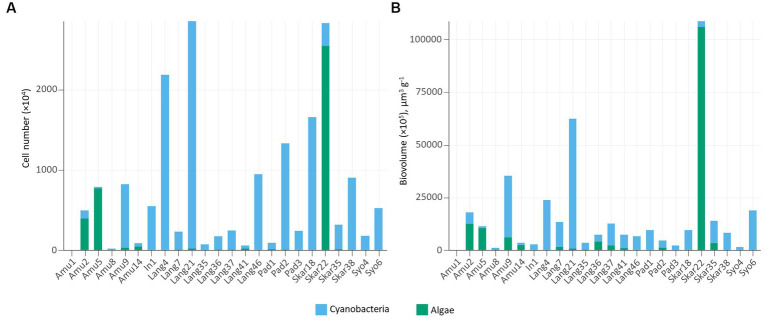
Number of cells **(A)** and cell biovolume **(B)** in the studied samples.

In addition, epifluorescence microscopy revealed the dominance of cyanobacteria in the majority of the samples (Figure 4). The highest cyanobacterial cell number and biovolume were recorded in Lang21. Eukaryotic microalgae prevailed in 4 samples (Amu2, Amu5, Amu14, and Skar22) and Skar22 had the highest cell number and biovolume. Furthermore, no eukaryotic algae were observed in 8 samples (Amu1, In1, Lang4, Pad3, Skar18, Skar38, Syo4, Syo6).

### Co-occurrence network

Co-occurrence analysis revealed 361 correlations within microbial community (Supplementary Figure S3; Supplementary Table S2). Of these, 272 co-occurrences were positive and 89 were negative. The majority of positive co-occurrences were between bacterial taxa, while the most of negative co-occurrences were observed between bacteria and eukaryotes.

Cyanobacteria co-occurred together with 29 bacterial (from Actinobacteriota, Armatimonadota, Bdellovibrionota, Chloroflexi, Firmicutes, Myxococcota, Patescibacteria, Planctomycetota, Proteobacteria and other Cyanobacteria) and 13 eukaryotic (from Amoebae, Alveolata, Chlorophyta, Fungi, and Amoebozoa) taxa. Furthermore, 4 negative co-occurrences were observed with bacteria (Bacillales/Chroococcales, Absconditabacteriales/Gloeobacterales, Bacteroidales/Nodosilineales, Micropepsales/Nodosilineales).

Eukaryotic algae had 18 and 13 co-occurrences with bacteria (including 3 with cyanobacteria) and eukaryotes, respectively. Four algal taxa (Chlorodendrophyceae, Coleochaetophyceae, Bacillariophyceae, and Zygnematophyceae) showed positive correlations with members of Alveolata, Amoebozoa, Fungi, Stramenopiles, and Rhizaria. Besides, algae co-occurred together with 7 bacterial (Acidobacteriota, Actinobacteriota, Armatimonadota, Bdellovibrionota, Firmicutes, Myxococcota, Proteobacteria) and 3 cyanobacterial (Chroococcales, Gloeobacterales, Synechococcales) taxa. In addition, 3 negative correlations were observed between algae and bacteria (Lactobacillales/Chlorodendrophyceae, Oligoflexales/Zygnematophyceae, Tissierellales/Klebsormidiophyceae).

## Discussion

### Overall diversity

The bacterial community in various terrestrial environments of East Antarctica was mainly represented by Chloroflexi, Cyanobacteria, and Firmicutes (Figure 2). These bacterial phyla are generally found in Antarctic terrestrial habitats (Doytchinov and Dimov, 2022). In contrast, Proteobacteria, Bacteroidetes, and Cyanobacteria are the primary inhabitants of soils and biocrusts in maritime Antarctica (Rippin et al., 2018; Bendia et al., 2023). The terrestrial bacterial community in the Sør Rondane Mountains, located in continental Antarctica, is mainly dominated by Proteobacteria, Bacteroidetes, and Deinococcus-Thermus (Obbels et al., 2016), while Actinobacteria prevail in soils from the McMurdo Dry Valleys (Mashamaite et al., 2023). Such discrepancies between the different parts of Antarctica could suggest that distance is an important parameter influencing microbial diversity.

Furthermore, Cyanobacteria were the most abundant phylum in the majority of the samples. Cyanobacterial rock colonization and/or biofilm formation in some samples (Amu9 and In1) is typical for the extreme environmental conditions of Antarctica (de Los Ríos et al., 2003). Furthermore, several biocrust samples (Amu14, Lang7, Lang37, Skar18, Syo4, Syo6) were dominated by Cyanobacteria. Biocrusts are considered as main vegetation in Antarctica and Cyanobacteria greatly contribute to their formation and development (Büdel and Colesie, 2014). However, the biocrusts collected from the rookeries (Amu1 and Skar19) were dominated by Firmicutes. These bacteria are involved in guano decomposition (Grzesiak et al., 2020) and were previously recorded in Antarctic ornithogenic soils colonized by penguins (Kim et al., 2012). They were also strongly correlated with total P and mineral N content. In addition, the endolithic and hypolithic samples (Lang46 and Skar38, respectively) were dominated by Chloroflexi.

The eukaryotic community in the Antarctic terrestrial samples was represented overall by Alveolata, Chloroplastida, Metazoa, and Rhizaria (Figure 2), consistent with findings from previous molecular studies (Obbels et al., 2016; Rippin et al., 2018). While different primer pairs may lead to under- or overrepresentation of certain microbial groups, the dominant taxa are consistently observed across all primer sets (Uddin et al., 2020). Biocrusts in this study were mainly dominated by Chloroplastida, which is typical for this ecosystem in Antarctica (Büdel and Colesie, 2014). However, Metazoa, in particular Tardigrada, prevailed in several biocrusts samples (Lang7, Lang21, Lang37, Syo6). This phylum, known as water bears, is resistant to various extreme conditions and has been observed in Antarctic soils (Kaczmarek et al., 2014). However, they do not favor ornithogenic soils (Smykla et al., 2012), which was confirmed in this study.

Co-occurrence network analysis revealed a higher number of positive correlations between microorganisms in the Antarctic terrestrial environment and most of these were between bacterial taxa (Supplementary Figure S3). Bacteria are known to live in communities and are capable of cooperating with each other (Stubbendieck et al., 2016). Similarly, there were a high number of positive correlations between bacteria and eukaryotes. For example, co-occurrence of microbial phototrophs and fungi was found between Zygnematophyceae and Blastocladiomycetes. Blastocladiomycota, previously classified as a group within Chytridiomycota, were identified in association with algae and various plants based on fossil evidence (Strullu-Derrien, 2016). Furthermore, a positive co-occurrence between Cryptomycota and Chlorodendrophyceae in the studied samples may indicate the formation of saprotrophic associations (Vass et al., 2022) between both taxa in Antarctic terrestrial environments.

### In-depth assessment of microbial phototrophs

Cyanobacterial community was dominated by filamentous cyanobacteria Oscillatoriales and Pseudanabaenales in the majority of the samples (Figure 2; Supplementary Table S1). These cyanobacteria are typical inhabitants of polar terrestrial environments and due to their motility and production of extracellular polymeric substances (EPS), they can adapt to the extreme environmental conditions (Tashyreva and Elster, 2012). Surprisingly, *Phormidium* and *Microcoleus* were absent from the samples, despite previous reports of their presence in Antarctica (Pushkareva et al., 2018; Rippin et al., 2018). This could potentially be attributed to the use of different primers or older database versions in the earlier studies. In addition, the black biocrusts or moss-containing samples were dominated by heterocystous cyanobacteria Nostocales. Several identified genera (*Nodularia*, *Nostoc*, *Calothrix*, and *Rivularia*) are able to epiphytically exist on the surface of moss, as previously reported in the polar regions (Solheim and Zielke, 2002). These genera provide fixed nitrogen to the moss and, in turn, receive a supply of carbohydrates and protection against desiccation and UV radiation (Stewart et al., 2011). Likewise, it has been previously shown that communities of Nostocales inhabit biocrusts in Antarctica (Pushkareva et al., 2018). Surprisingly, no cyanobacteria from order Nostocales were detected in the green sand sample (Skar35). In temperate regions, the diversity of cyanobacteria in sand is lower than in biocrusts, although heterocystous cyanobacteria are still present (Glaser et al., 2022). Besides, black sand sample (Pad3) contained several Nostocales genera. In addition, 47% of cyanobacterial reads in the hypolithic sample (Skar38) belonged to ASV26, assigned to *Chroococcidiopsis*. This genus has been previously detected in Antarctic hypoliths (Van Goethem et al., 2016). Similarly, ASV35, assigned to the order level of Nostocales, constituted 90% of cyanobacterial reads in the endolithic sample (Lang46), which is consistent with previous research (De Los Ríos et al., 2014). These cyanobacteria frequently prevail in lithic microbial communities in Antarctica alongside Chloroflexi, as observed in this study.

The studied samples exhibited a dominance of Chlorophyceae, Trebouxiophyceae, Ulvophyceae, Bacillariophyceae, and Zygnematophyceae within the algal community (Figure 2; Supplementary Table S1). These eukaryotic algae are frequently found in Antarctica’s terrestrial surroundings (Obbels et al., 2016; Rybalka et al., 2023). Furthermore, algae from order Prasiolales (Trebouxiophyceae) were detected in all samples and were particularly dominant in the samples from seal mummy and penguin feather surfaces (Skar22 and Amu5, respectively), as well as from penguin rookeries (Amu1, Skar19). These green algae typically inhabit environments rich in organic matter content and have been found in the Victoria Land, Princess Elizabeth Land and islands around the Antarctic Peninsula (Moniz et al., 2012; Sharma et al., 2020). In addition, the ASVs assigned to Xanthophyceae were mainly from the order Mischococcales, specifically the genera *Botrydiopsis*, *Chlorellidium* and *Pleurochloris*. Some of the identified species, namely *Pseudopleurochloris antarctica* and *Chlorellidium tetrabotrys* have previously been documented in pack-ice (Andreoli et al., 1999), soils and terrestrial environments (Broady, 1976) of Antarctica, respectively.

Cyanobacterial abundance and biovolume measured by epifluorescence microscopy prevailed in the majority of the samples (Figure 4). These trends are consistent with previous observations in Arctic biocrusts (Pushkareva et al., 2017, 2022). Cyanobacteria are the primary producers and often among the first colonizers of barren areas (Nemergut et al., 2007). In contrast, eukaryotic algae favor more developed environments with high organic matter content (Kaštovská et al., 2005). In this respect, eukaryotic algae dominated samples, either on the surface of seal mummy (Skar22) or penguin feathers (Amu5), where *Prasiola* was observed, or in biocrusts with penguin feathers (Amu14) or near the rookery (Amu2).

## Conclusion

This study reveals high heterogeneity of both prokaryotic and eukaryotic microbial communities in various terrestrial environments of East Antarctica. The dominant bacterial phyla observed in the samples were Chloroflexi, Cyanobacteria, and Firmicutes, while the dominant eukaryotic taxa were Chloroplastida, Alveolata, Rhizaria, and Metazoa. Cyanobacteria and eukaryotic microalgae were particularly abundant in the majority of the samples. The microbial community composition and diversity were influenced by the site of origin, indicating spatial variation in microbial populations. In addition, we observed several positive co-occurrences between microphototrophs and fungi, indicating that these organisms might promote each other’s growths. However, further studies are needed to investigate the specific nature of interactions between these microorganisms.

In conclusion, this study enhances our understanding of species diversity and distribution in East Antarctica and serves as a valuable baseline for future research involving metagenomic and metatranscriptomic analyses in this region.

## Data availability statement

The datasets presented in this study can be found in online repositories. The names of the repository/repositories and accession number(s) can be found in the article/Supplementary material.

## Author contributions

EP: Conceptualization, Data curation, Writing – original draft, Writing – review & editing. JE: Conceptualization, Writing – review & editing. SK: Writing – review & editing. SI: Writing – review & editing. BB: Conceptualization, Writing – review & editing.
