# Supplementary material for: Microbial community composition of terrestrial habitats in East Antarctica with a focus on microphototrophs
Source: Front Microbiol. 2024 Jan 5;14:1323148. doi: 10.3389/fmicb.2023.1323148 (PMC10797080; doi:10.3389/fmicb.2023.1323148)
Supplement: Supplementary file 2 [file Data_Sheet_1.docx]

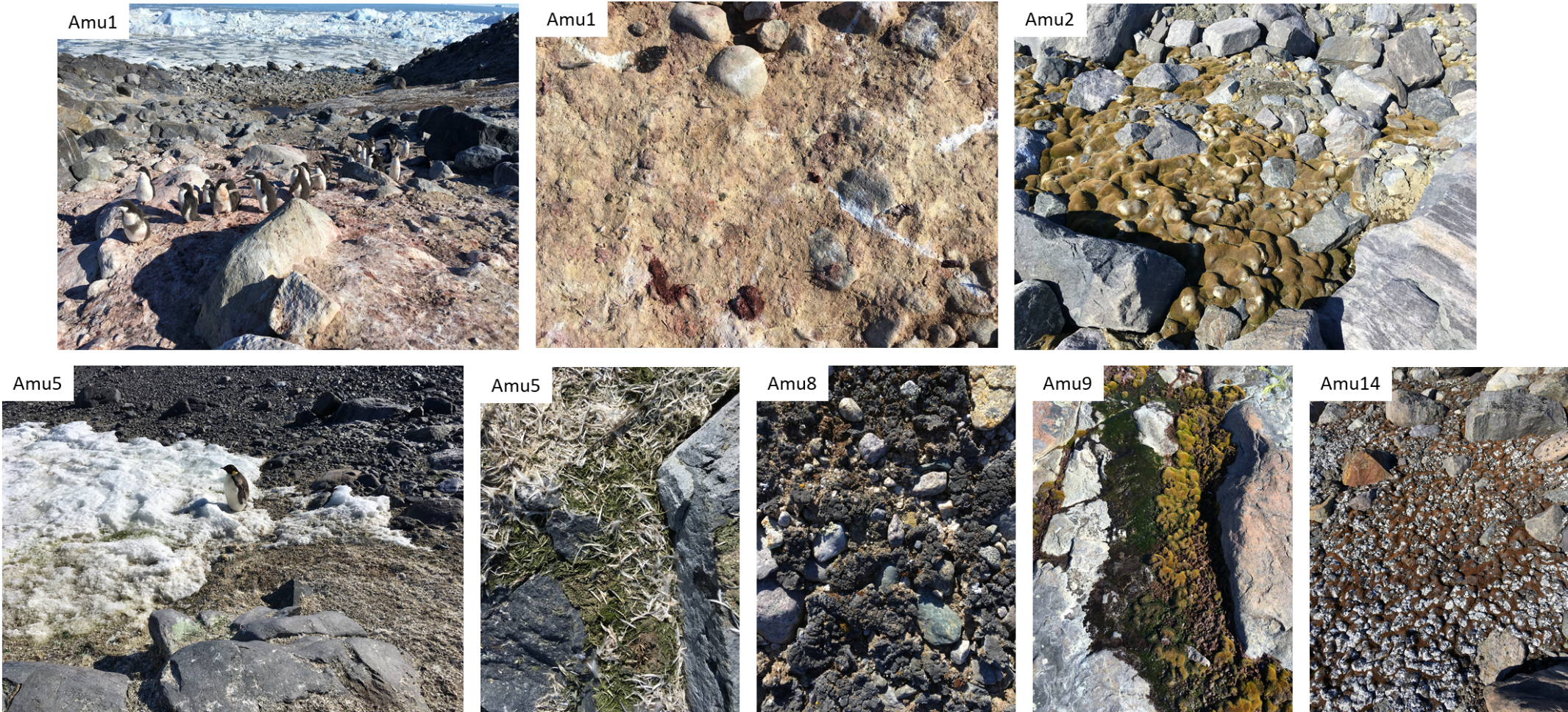


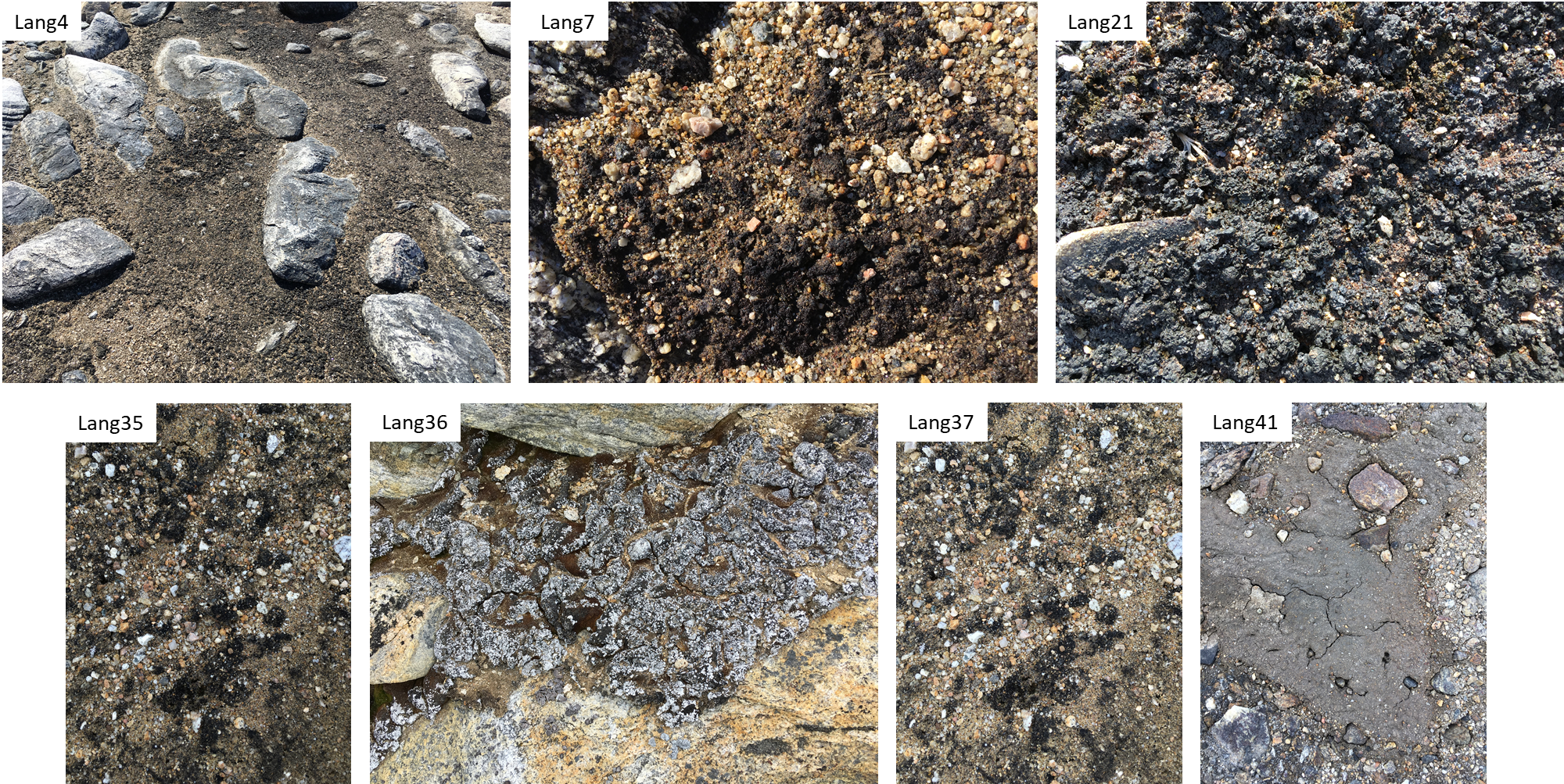


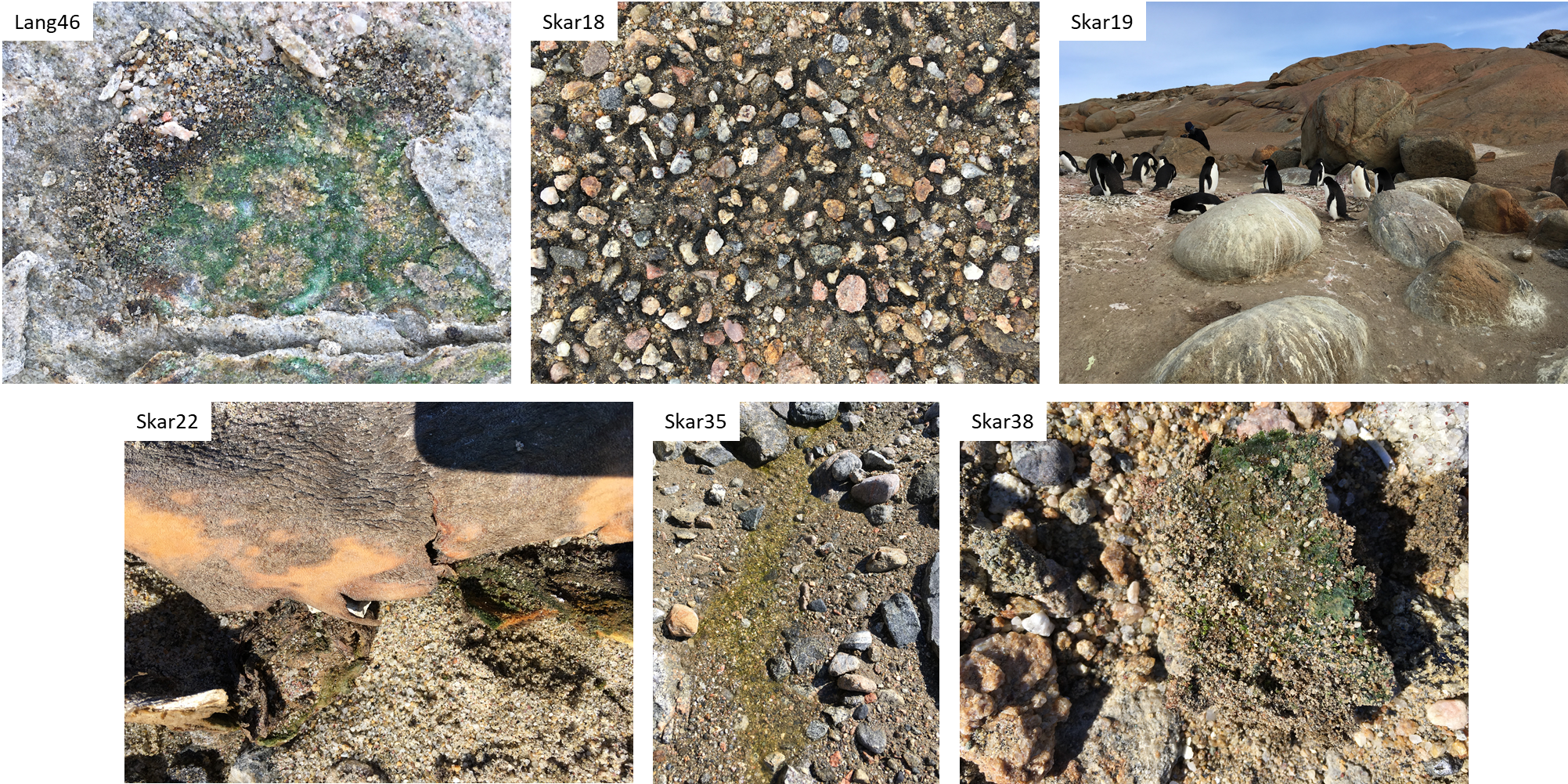


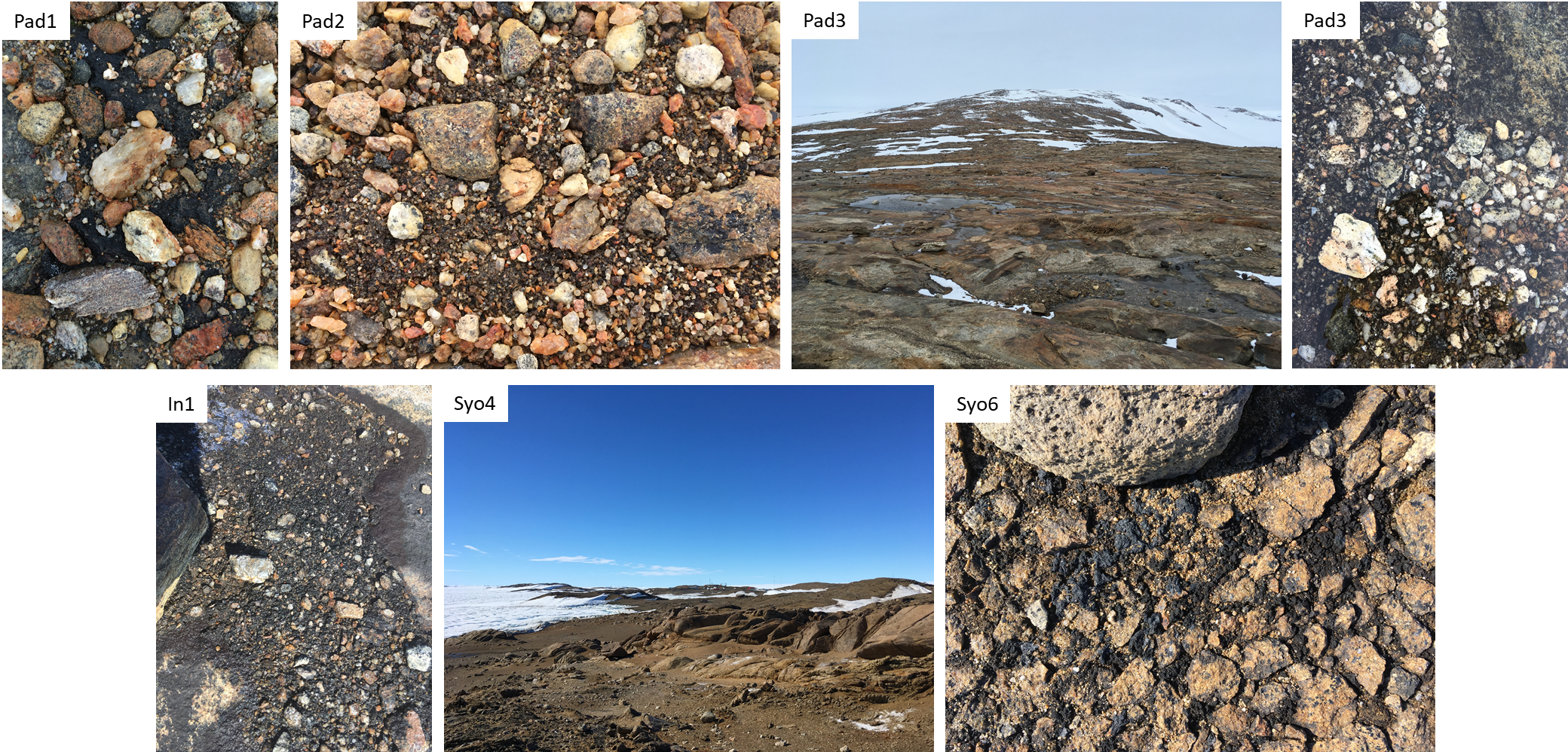


**Supplementary Figure 1** – Photos of the collected samples from Antarctica.


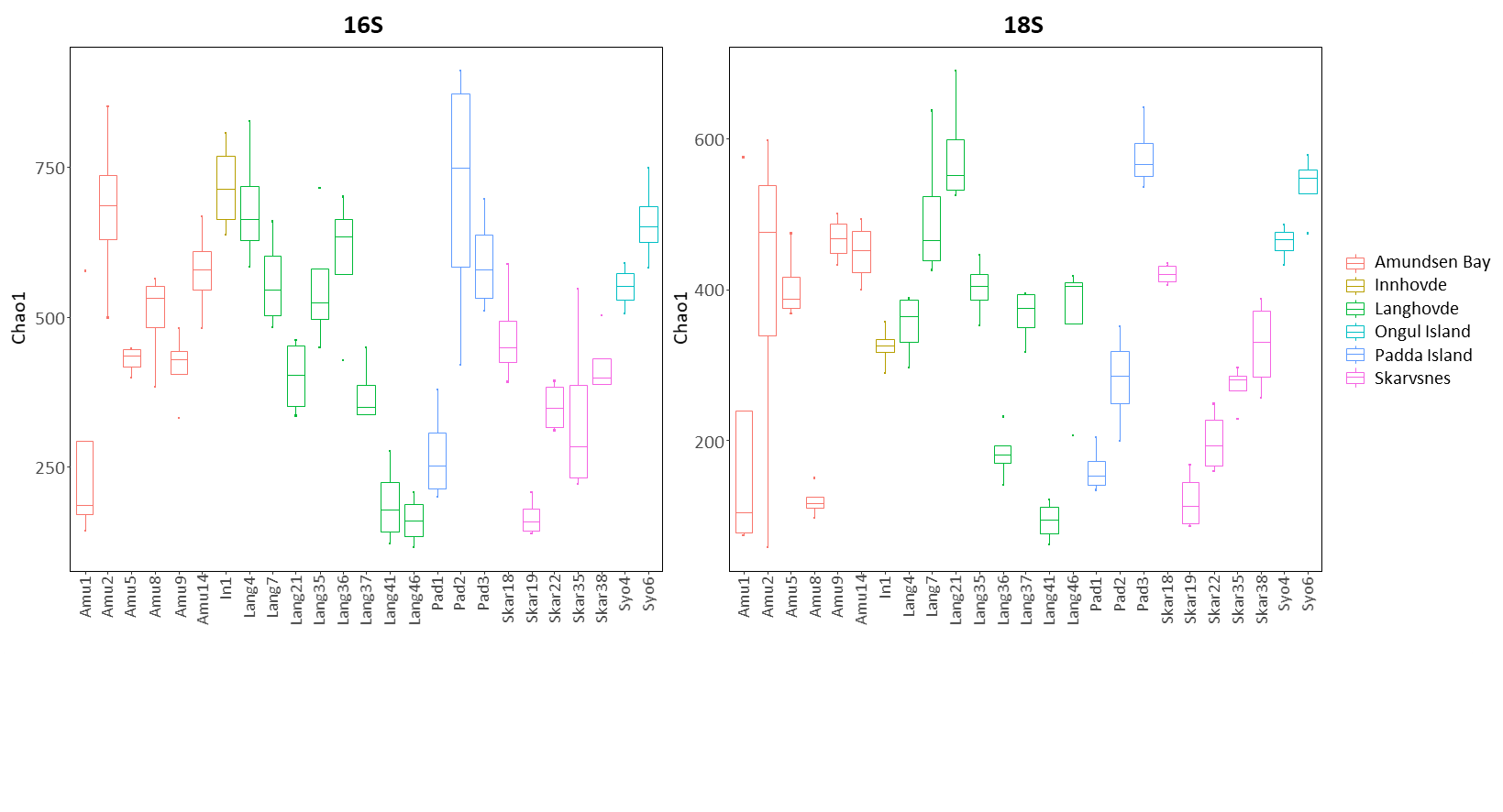


**Supplementary Figure 2** – Chao1 diversity index in studied samples: (16S) – bacteria and (18S) – eukaryotes. Boxes represent the interquartile range and the horizontal line inside the box defines the median

a.


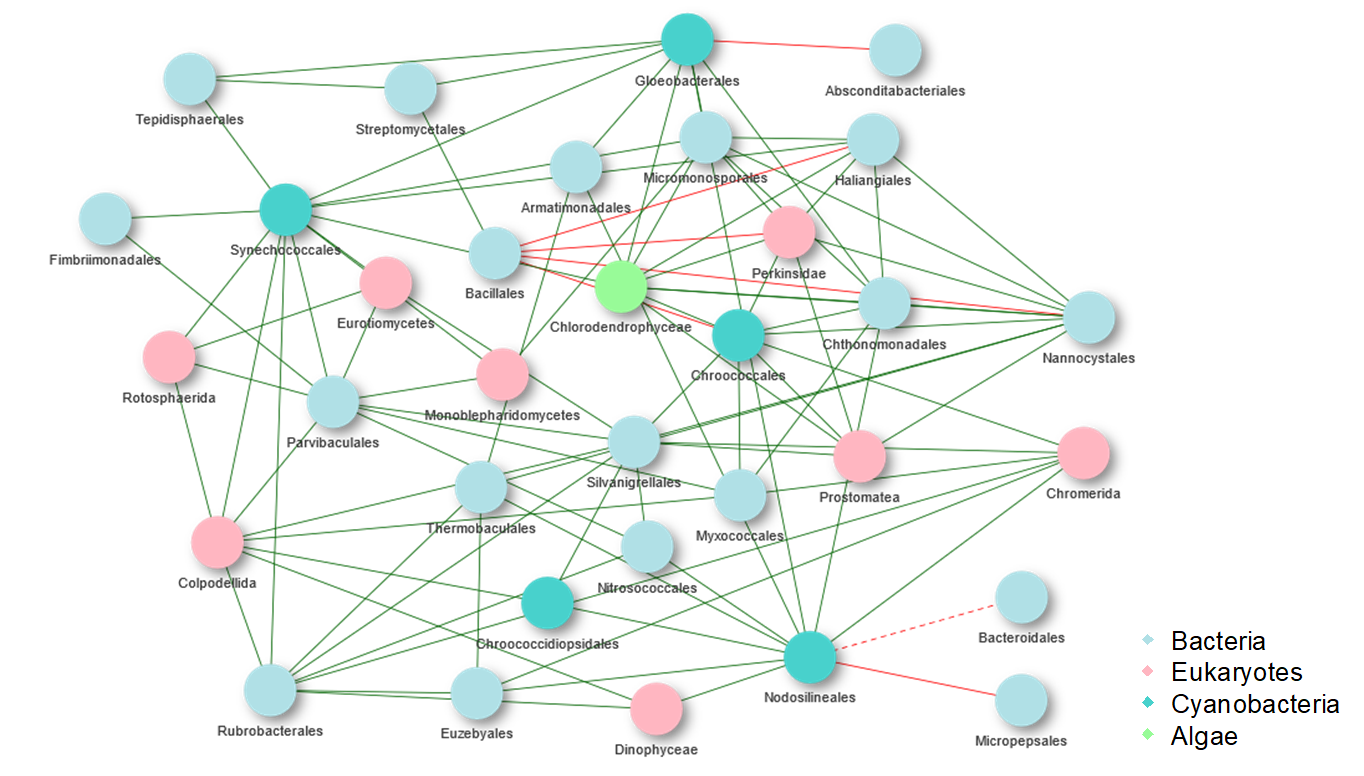


b.


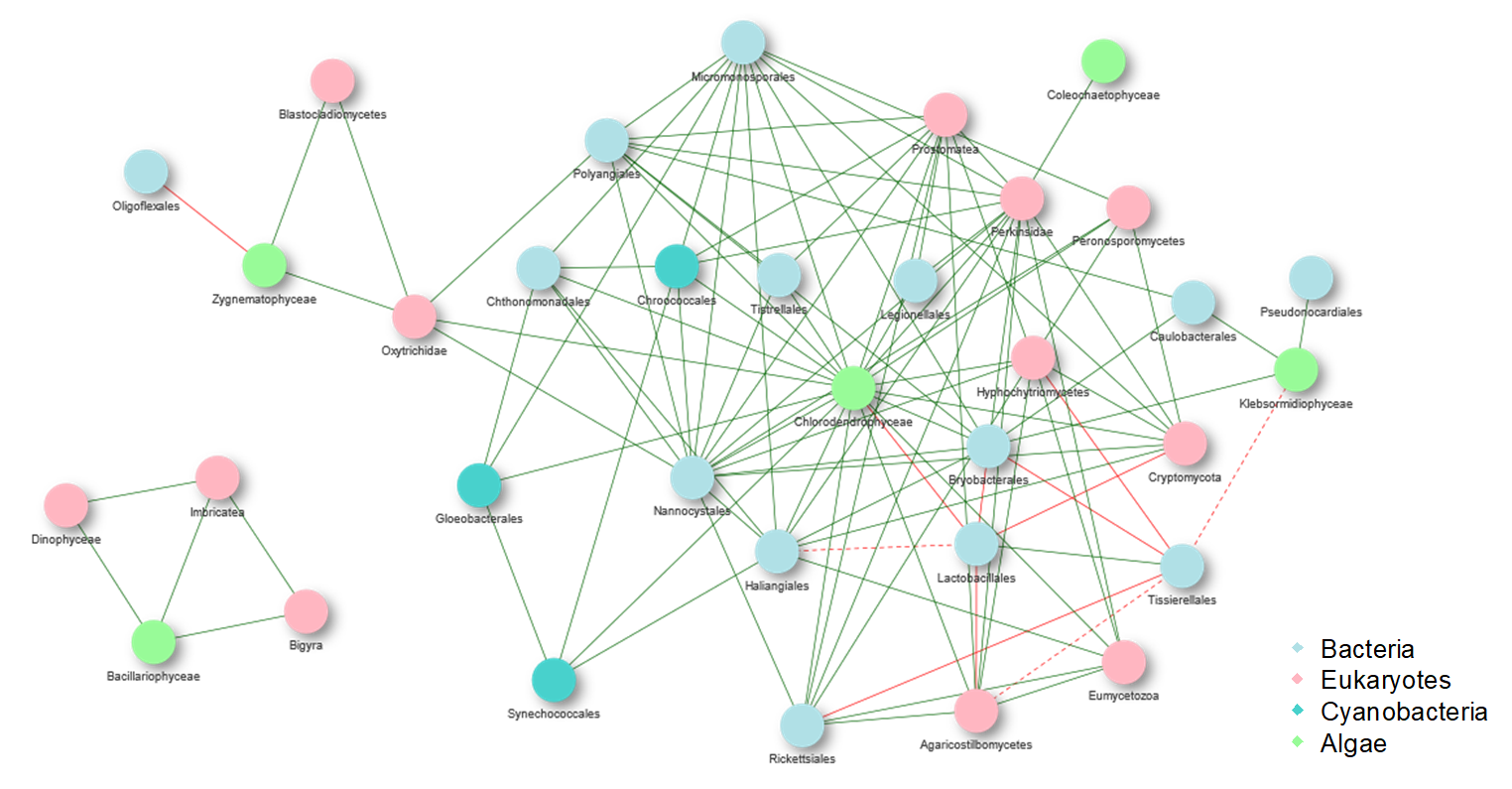


**Supplementary Figure 3** – Co-occurrence networks of cyanobacteria (a) and eukaryotic microalgae (b). Green and red lines indicate positive and negative correlations, respectively.
